# Application-matched point-of-care 3D printing of functionalized polyetheretherketone (PEEK) composites for patient-specific craniomaxillofacial implants

**DOI:** 10.1186/s41205-026-00331-z

**Published:** 2026-06-24

**Authors:** Jokin Zubizarreta Oteiza, Yannick Krieger, Daniel Seiler, Florian M. Thieringer, Neha Sharma

**Affiliations:** 1 Medical Additive Manufacturing Research Group (Swiss MAM), Department of Biomedical Engineering, University of Basel, Hegenheimermattweg 167b, Allschwil, 4123 Switzerland; 2 Clinic of Oral and Cranio-Maxillofacial Surgery and 3D Print Lab, University Hospital Basel, Petersgraben 4, Basel, 4031 Switzerland; 3 3DSystems, Flößergasse 4, 81369 Munich, Germany; 4 Institute for Medical Engineering and Medical Informatics, University of Applied Sciences and Arts Northwestern Switzerland, Hofackerstrasse 30, Muttenz, 4132 Switzerland; 5 Duke-NUS Medical School, 8 College Road, Singapore, 169857 Singapore

**Keywords:** 3D printing, Additive manufacturing, Barium sulfate, Bioactive polymers, Biphasic calcium phosphate, Carbon fiber reinforcement, Radiopaque polymers, Point of care, Thermoplastic composites

## Abstract

**Background:**

Native polyetheretherketone (PEEK) exhibits limitations in mechanical strength, radiographic visibility, and bioactivity for craniomaxillofacial (CMF) applications. This proof-of-concept study establishes the feasibility of point-of-care (POC) manufacturing for three functionalized PEEK composites for patient-specific CMF implants using a high-temperature material extrusion (MEX) system, with application-specific material selection to overcome these limitations.

**Methods:**

Carbon fiber-reinforced PEEK (CFR-PEEK), barium sulfate-filled PEEK (BaSO₄-PEEK), and biphasic calcium phosphate-filled PEEK (BCP-PEEK) were fabricated into patient-specific mandibular reconstruction plates, orbital floor implants, and chin augmentation implants, respectively. Manufacturing success rates, dimensional accuracy via root mean square (RMS) deviation analysis, post-sterilization dimensional stability, and layer adhesion quality were evaluated.

**Results:**

Fabrication success rates of 100% (CFR-PEEK), 100% (BaSO₄-PEEK), and 85.7% (BCP-PEEK) were achieved. Dimensional accuracy analysis revealed RMS deviations of 0.16–0.29 mm between 3D-printed implants and original designs, within clinically acceptable ranges. Post-sterilization dimensional changes were minimal (RMS 0.05–0.07 mm). Complete layer adhesion was demonstrated across all materials, with no delamination or cracking observed.

**Conclusion:**

These findings establish the manufacturing viability of POC fabrication of application-matched PEEK composites for patient-specific CMF implants, enhancing mechanical, radiographic, and bioactive properties whilst maintaining geometric customization.

## Introduction

The convergence of personalized medicine and digital manufacturing technologies is fundamentally changing the production of patient-specific implants (PSI). Among high-performance polymers suitable for long-term implantation, polyetheretherketone (PEEK) has emerged as a promising biomaterial due to its unique combination of biocompatibility, radiolucency, and mechanical properties that closely resemble those of cortical bone [1–3]. These characteristics have led to widespread use in spinal cages, craniomaxillofacial (CMF) reconstruction, orthopedic implants, and dental prosthetics [4, 5]. However, as clinical demands evolve and reconstructive challenges become increasingly complex, bioinert (unfilled) PEEK faces critical limitations that limit its clinical utility.

While PEEK’s intrinsic radiolucency benefits specific applications, such as postoperative tumor surveillance, it severely limits implant visualization during surgery and during follow-up imaging when direct implant monitoring is required [6]. Additionally, although PEEK’s bone-mimetic mechanical properties reduce stress shielding compared with metallic alternatives [7], they may be insufficient at high-load anatomical sites. Perhaps most significantly, PEEK’s biological inertness and hydrophobic surface characteristics inhibit cell adhesion and osseointegration, compromising long-term implant integration [8, 9].

These limitations have catalysed the development of functionalized PEEK composites engineered to address specific clinical deficiencies. Carbon fiber-reinforced PEEK (CFR-PEEK) incorporates short carbon fibers to substantially enhance stiffness and load-bearing capacity for demanding reconstructive applications [10, 11]. Barium sulfate-filled PEEK (BaSO₄-PEEK) improves radiopacity, enabling intraoperative placement verification and postoperative monitoring through conventional medical imaging [12]. Biphasic calcium phosphate-filled PEEK (BCP-PEEK) incorporates the principal mineral component of natural bone to introduce bioactive properties that promote osseointegration and direct bone apposition [13, 14].

Despite commercial availability and extensive characterization of bulk materials, a critical gap remains: the processability of these composites into complex, patient-specific CMF geometries using hospital-based additive manufacturing (AM) systems has not been demonstrated. This manufacturing feasibility gap fundamentally limits clinical translation, as point-of-care (POC) production requires the reliable processing of anatomically intricate structures within clinically relevant timeframes.

AM, particularly material extrusion (MEX) technology, enables on-demand production of PSI directly from medical imaging data [15, 16]. Hospital-based manufacturing offers distinct advantages, such as eliminating supply chain delays, providing immediate availability for emergency reconstruction, and enabling cost-effective production for complex cases [17, 18]. Under Article 5(5) of the European Union Medical Device Regulation 2017/745 (EU MDR), hospitals may manufacture patient-specific devices in-house, provided rigorous quality and safety protocols are maintained [19] and the general safety and performance requirements (GSPR) set out in Annex I of the EU MDR are met. Previous work has demonstrated the POC of three-dimensional (3D) printed PEEK cranial implants using regulation-compliant hospital digital manufacturing workflows [20, 21]. However, whether functionalized PEEK composites with abrasive fillers, bioactive agents, and radiopaque materials can be successfully printed into complex CMF geometries using POC AM infrastructure remains unknown.

The objective of this proof-of-concept study is to establish the manufacturing feasibility of three functionalized PEEK composites for patient-specific CMF applications using a high-temperature MEX system suitable for hospital deployment. This study employs an application-specific material selection paradigm: CFR-PEEK for mandibular reconstruction plates requiring enhanced mechanical reinforcement; BaSO₄-PEEK for orbital implants requiring radiographic visibility; and BCP-PEEK for chin augmentation implants requiring bone-implant integration. Optimized processing parameters, dimensional accuracy assessment, and complete design-to-part workflows are established for each composite geometry combination.

The material-application pairing in this study is a preliminary, hypothesis-driven framework based on material properties and clinical experience. Reconstruction plates are subjected to high masticatory loads, supporting the use of mechanically reinforced CFR-PEEK [10, 11]. While orbital implants require precise positioning in anatomically constrained regions where imaging-based verification of implant placement is essential, supporting the use of radiopaque BaSO₄-PEEK [12]. Lastly, onlay-type augmentation implants benefit from improved bone-implant integration, providing long-term positional stability and supporting the use of bioactive BCP-PEEK [13, 14]. These pairings are not exclusive, and further studies are needed to validate their functional performance in clinical settings.

By establishing manufacturing feasibility and dimensional accuracy for complex patient-specific geometries, this study provides the technical foundation for POC production of application-matched PEEK composites in CMF reconstruction.

## Materials and methods

### Materials

**Fig. 1:**
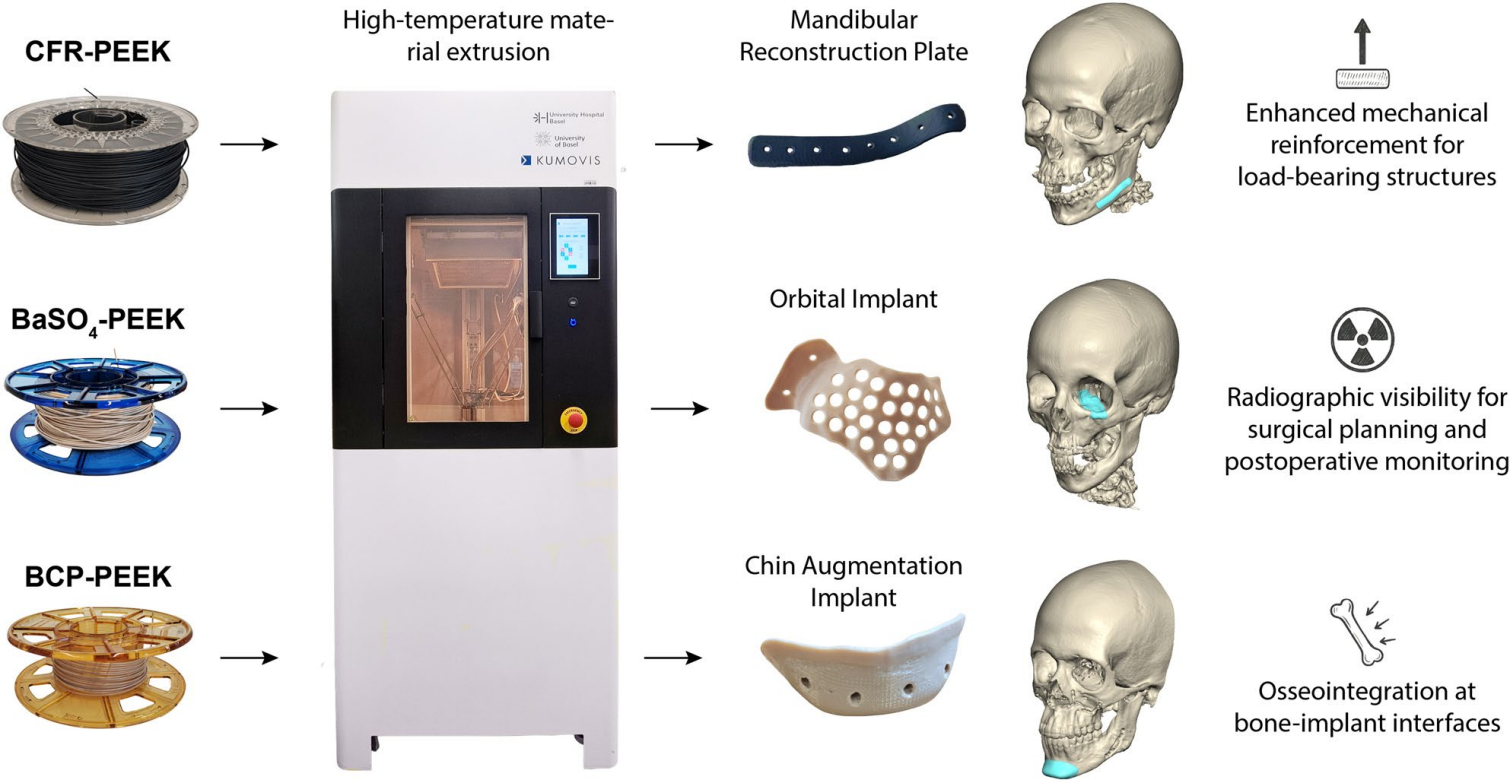
Application-matched material selection strategy for patient-specific craniomaxillofacial (CMF) implants. Three functionalized PEEK composites were fabricated via a high-temperature material extrusion (MEX) into application-matched implant geometries: Carbon Fiber-Reinforced PEEK (CFR-PEEK) for mandibular reconstruction plates (enhanced mechanical reinforcement for load-bearing structures), Barium Sulfate-Filled PEEK (BaSO₄-PEEK) for orbital floor implants (radiographic visibility for surgical planning and postoperative monitoring), and Biphasic Calcium Phosphate-filled PEEK (BCP-PEEK) for chin augmentation implants (osseointegration at bone-implant interfaces)

Three functionalized PEEK composite filaments were evaluated in this proof-of-concept study, each selected to address distinct functional requirements in CMF reconstruction (Fig. 1; Table 1). All filaments were supplied at a nominal diameter of 1.75 mm and stored in vacuum-sealed packaging with desiccant. Before 3D printing, filaments were dried in a convection oven (UF30, Memmert GmbH, Schwabach, Germany) at 100 °C for at least 12 h to reduce moisture content to below 0.02 wt%, as recommended by the manufacturer for PEEK production.

**Table 1:** PEEK composite materials and specifications

Material	Product Code	Manufacturer	Filler Content	Functional Role
CFR-PEEK	VESTAKEEP^®^ iC4612 3DF	Evonik Industries AG, Essen, Germany	12% short carbon fibers	Enhanced mechanical reinforcement for load-bearing structures
BaSO₄-PEEK	VESTAKEEP^®^ iC4520 3DF	Evonik Industries AG, Essen, Germany	20% barium sulfate	Radiographic visibility for surgical planning and postoperative monitoring
BCP-PEEK	VESTAKEEP^®^ iC4800 3DF	Evonik Industries AG, Essen, Germany	10–20% biphasic calcium phosphate	Osseointegration at bone-implant interfaces

Carbon Fiber−Reinforced PEEK (CFR−PEEK); Barium Sulfate−Filled PEEK (BaSO₄−PEEK); Biphasic Calcium Phosphate−filled PEEK (BCP−PEEK)

### Implant design and digital preparation

**Fig. 2:**
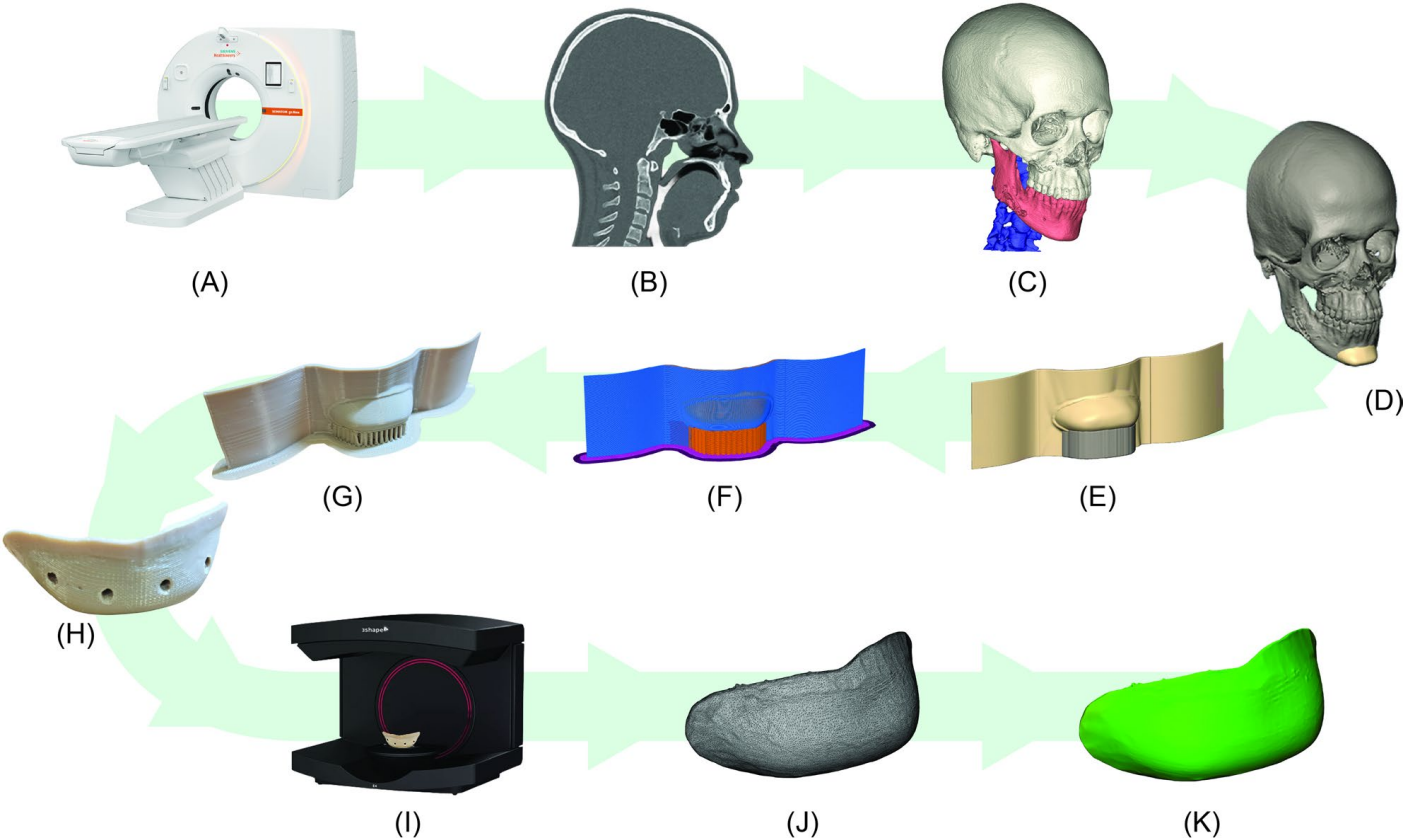
Digital workflow for the manufacturing of a patient-specific implant via material extrusion (MEX). (**A**) acquisition of computed tomography (CT) data, (**B**) import of medical imaging datasets, (**C**) anatomical segmentation, (**D**) virtual surgical planning, (**E**) computer-aided-design (CAD) of patient-specific implant with support structures, (**F**) slicing for toolpath generation, (**G**) 3D printing of the implant, (**H**) implant after post-processing, (**I**) optical scanning of the fabricated part, (**J**) mesh generation and analysis, and (**K**) part-to-CAD comparison for dimensional verification

The complete digital workflow for patient-specific implant manufacturing is illustrated in Fig. 2. Anonymized computed tomography (CT) data from a healthy adult patient (slice thickness 0.8 mm) were segmented and reconstructed using medical image processing software (Materialise Mimics Innovation Suite v26, Materialise NV, Leuven, Belgium) to generate a 3D CMF model (Fig. 2A, B and C). Three clinically representative implant types were designed using computer-aided design (CAD) software (Geomagic Freeform Plus V2022.0.34, Hexagon AB, Stockholm, Sweden) (Fig. 2D), each selected to evaluate distinct geometric complexities and functional requirements (Table 2). Design specifications included integrated support structures (Fig. 2E) and post-processing access features based on anticipated material-specific processing requirements. All geometries were exported as Standard Tessellation Language (STL) files for manufacturing. Six replicate implants were fabricated for each design type (*n* = 18 total) to evaluate manufacturing feasibility and dimensional accuracy.

The digital workflow applied in this study follows established protocols for patient-specific implant design in CMF surgery, including CT-based segmentation, virtual surgical planning, and CAD-based implant modelling, which have been previously described across both metallic and polymeric patient-specific devices [22–24], including POC PEEK implant fabrication [15, 20, 21]. All implant designs were performed by a biomedical engineer (PhD in Biomedical Engineering) with over three years of clinical engineering experience, including the design and manufacture of more than 50 patient-specific PEEK implants for routine clinical use.

Several design features were adapted specifically to accommodate high-temperature PEEK MEX constraints. Support structure geometries were simplified to minimize heat retention and facilitate clean manual removal while avoiding contact with anatomically critical bone-facing surfaces, in accordance with established principles for AM medical devices [25, 26]. Screw hole and drainage channel features were incorporated as printed guide features and finalized by post-print drilling rather than direct printing, as the dimensional tolerance achievable via MEX was considered insufficient for surgical screw engagement and drainage channel patency. Wall thicknesses in the orbital implant were maintained at a minimum of 0.8 mm to ensure reliable single-pass deposition.

**Table 2:** Application-specific craniomaxillofacial (CMF) implant designs

Implant Type	Material	Key Design Features	Material Selection Rationale
Mandibular Reconstruction Plate	CFR-PEEK	Complex double-curved geometry spanning 60–80 mm; integrated screw holes (Ø3 mm, ± 0.2 mm tolerance); uniform 2.5 mm thickness; bounding box: 81 × 15 × 19 mm; volume: 2,323 mm³; mass: 3.25 g	Enhanced stiffness required for load-bearing function; evaluates processability of fiber-reinforced composite in spanning structural geometry
Orbital Implant	BaSO₄-PEEK	Thin-walled structure (0.8 mm) with double-curved geometry; drainage channels (Ø2–3 mm); precise anatomical surface matching; bounding box: 48 × 27 × 12 mm; volume: 847 mm³; mass: 1.37 g	Radiographic visibility essential for surgical positioning; evaluates the printability of radiopaque filler in sub-millimetre wall thickness
Chin Augmentation Implant	BCP-PEEK	Variable thickness gradient (7 mm apex to 0.6–0.8 mm periphery); internal porous architecture (0.2–0.5 mm pore size); dual-surface topology; bounding box: 44 × 18 × 19 mm; volume: 2,871 mm³; mass: 4.16 g	Bioactive interface needed for osseointegration; evaluates processing of ceramic filler with variable cross-section and porous features

Carbon Fiber−Reinforced PEEK (CFR−PEEK); Barium Sulfate−Filled PEEK (BaSO₄−PEEK); Biphasic Calcium Phosphate−filled PEEK (BCP−PEEK)

### Additive manufacturing

The STL files were imported into slicing software (Simplify3D, v4.1.2, Cincinnati, Ohio, USA) for toolpath generation (Fig. 1F). Printing parameters for each composite were established through systematic optimization trials (*n* = 3–5 iterations per material), with successful prints defined as complete part fabrication without delamination, warping > 2 mm, or nozzle clogging. Temperature ranges were selected based on manufacturer recommendations and refined through direct observation of layer adhesion quality. Print speeds were adjusted to account for filler-specific rheological properties, with fiber-reinforced composites requiring higher speeds to prevent fiber-orientation artifacts.

**Fig. 3:**
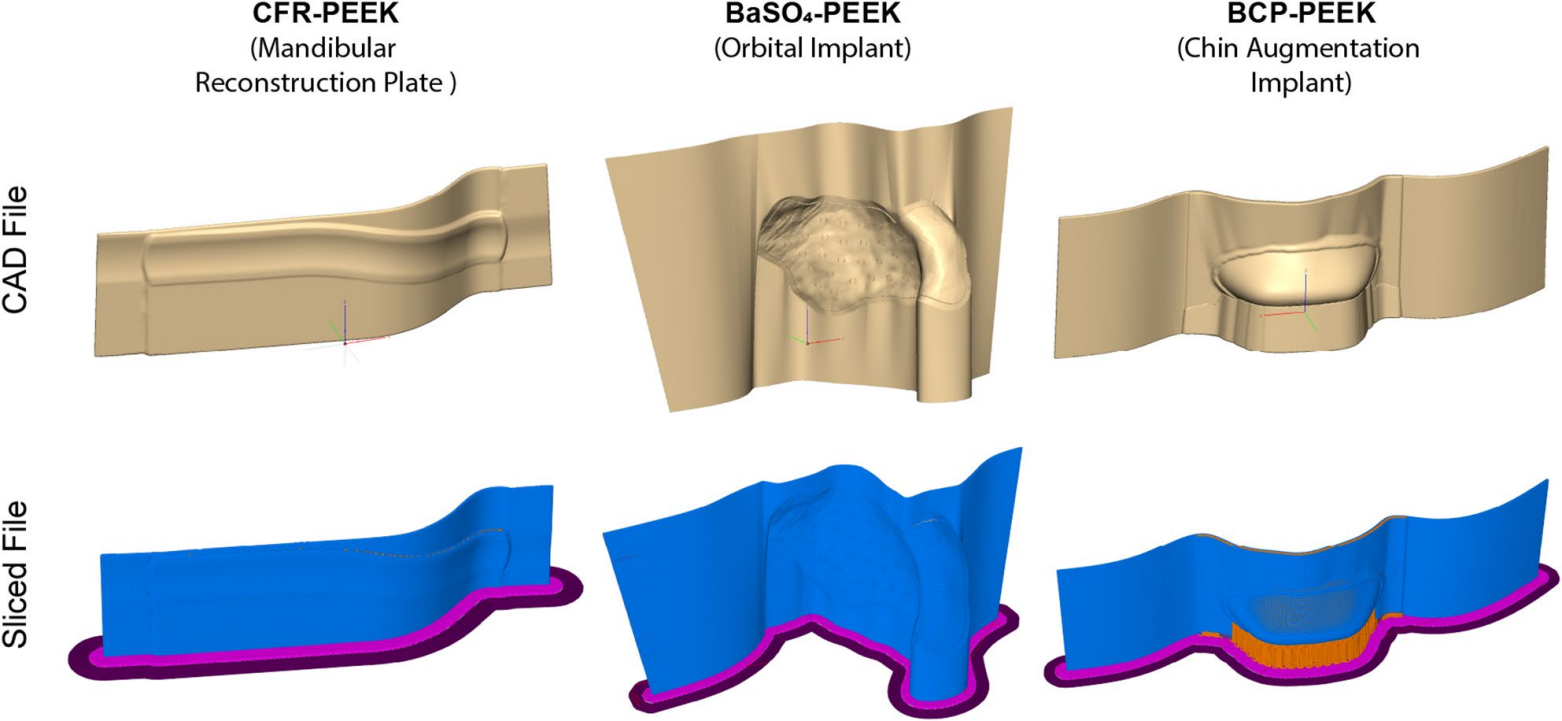
Representative slicing workflow for patient-specific implants. Top row (CAD files): original implant geometries with support structures for (**A**) CFR-PEEK mandibular reconstruction plate, (**B**) BaSO₄-PEEK orbital implant, and (**C**) BCP-PEEK chin augmentation implant. Bottom row (sliced files): corresponding build preparation views illustrating print orientation and support structures for each design

Build orientation was selected based on three primary criteria consistent with previously reported design approaches for AM of patient-specific CMF medical devices [25, 27]: minimizing support contact with anatomically critical bone-facing surfaces; optimizing surface quality on patient-contact faces; and enabling the most continuous possible nozzle toolpath across the geometry. The mandibular plate was oriented horizontally on the build platform to maximize layer continuity across its spanning curved geometry and to ensure uniform layer adhesion along the primary load-bearing axis. The orbital implant was oriented vertically, with the concave bone-facing surface positioned away from support contact to preserve anatomical surface accuracy on the critical fitting surface. The chin implant was oriented at an angle to balance overhang minimization with continuous deposition across its variable-thickness profile (Fig. 3).

Support structures were designed as thin continuous wall enclosures fully surrounding the implant geometry, rather than conventional point or lattice supports (Fig. 3). This approach serves two primary purposes specific to high-temperature PEEK extrusion: first, the enclosing wall structure promotes uniform heat distribution across the entire build, reducing thermal gradients that would otherwise cause warping, shrinkage, or delamination in semi-crystalline PEEK composites; second, it enables a single continuous nozzle toolpath with minimal travel movements throughout the build. In PEEK MEX, interruptions to the continuous flow such as those caused by printed holes or isolated geometric features can create localized printing artifacts foci that act as preferential sites for delamination. The continuous wall enclosure strategy therefore directly addresses the delamination susceptibility inherent to high-temperature PEEK composites, while simultaneously improving dimensional stability and surface quality on implant-facing surfaces. The final optimized parameters are presented in Table 3.

**Table 3:** Printing parameters for the functionalized PEEK composite materials

Parameter	CFR-PEEK	BaSO₄-PEEK	BCP-PEEK
Nozzle Temperature	440–450 °C	420–430 °C	415–440 °C
Bed Temperature	270 °C	250 °C	240 °C
Chamber Temperature	230 °C	230 °C	230 °C
Nozzle Diameter	0.4 mm	0.4 mm	0.4 mm
Perimeter Shells	1	1	1
Outline Direction	Outside-In	Outside-In	Outside-In
Print Speed	3100 mm/min	1000 mm/min	1500 mm/min
Layer Height	0.18 mm	0.26 mm	0.28 mm
Infill Pattern	Rectilinear	Rectilinear	Rectilinear
Infill Density	100%	100%	80–100%*

Carbon Fiber−Reinforced PEEK (CFR−PEEK); Barium Sulfate−Filled PEEK (BaSO₄−PEEK); Biphasic Calcium Phosphate−filled PEEK (BCP−PEEK). * Infill density for BCP−PEEK varied between 80–100% depending on porous architecture requirements in bone−contacting regions

Implants were fabricated using a high-performance MEX system (EXT 220 MED, 3D Systems, Rock Hill, South Carolina, USA), specifically designed for high-temperature thermoplastic polymers (Fig. 1G). The system features a build volume of Ø220 × 160 mm, a layer resolution of 0.1–0.3 mm, and processes 1.75 mm-diameter filaments. Temperature control capabilities include nozzle temperatures up to 500 °C, build plate temperatures up to 300 °C, and chamber temperatures up to 250 °C. The system operates on a delta-kinematic architecture with three parallel arms that control print head movement, reducing inertial effects and enabling precise positioning. The heated build chamber provides laminar airflow for a homogeneous temperature distribution, with integrated HEPA filtration that meets International Organization for Standardization (ISO) 14,644 Class 7 standards.

All composites were extruded through a 0.4 mm-diameter tungsten carbide nozzle to minimize wear from abrasive fillers. Filaments were fed directly from the drying oven (maintained at 100 °C) to maintain dry conditions throughout printing. A heated stainless-steel build plate was secured via automatic vacuum-seal integration. The printer was preheated for 2.5 h before each use to achieve thermal equilibrium. Between material transitions, the nozzle was replaced, and all contact surfaces were cleaned with 99% isopropanol (3× wipe protocol) to prevent cross-contamination. Build plate calibration and nozzle purging routines (approximately 50 mm extrusion) were performed before printing each implant.

Support structures were removed using a precision micromotor handpiece system (Perfecta 600, W&H Dentalwerk Bürmoos GmbH, Salzburg, Austria) with carbide cutting discs and grinding tools of various diameters to remove residual support material and refine implant contours (Fig. 2H). Fixation holes and drainage channels were incorporated as-printed guide features and finalized by post-print drilling using tungsten carbide drills (1–3 mm diameter) guided by 3D-printed jigs, rather than being directly printed into the geometry. This approach was adopted for two reasons specific to the extrusion of high-temperature PEEK. First, printed holes interrupt the continuous nozzle toolpath that is essential for consistent interlayer adhesion in PEEK composites, hole boundaries introduce travel movements that create localized printing artifacts foci and preferential delamination sites. Second, the dimensional tolerance achievable for small circular features via MEX is insufficient for surgical screw engagement, where precise hole geometry is critical to ensure correct screw fit and avoid excessive mechanical stress on the surrounding implant material or compromised insertion accuracy in preoperatively planned trajectories near sensitive anatomical structures. Post-processing guides were fabricated from polylactic acid filament (Matte Ivory White, Bambu Lab, Shenzhen, China) using MEX (Bambu Lab X1 Carbon, Bambu Lab, Shenzhen, China) and resin (BioMed Clear, Formlabs, Massachusetts, USA) using low-force stereolithography (SLA) (Form 4B, Formlabs, Massachusetts, USA) 3D-printers to assist accurate positioning and angulation of drilled holes.

After post-processing, all implants were steam-sterilized in an autoclave at 134 °C for 18 min at 2.1 bar. Dimensional measurements were performed both before and after sterilization to assess dimensional stability and thermal effects.

### Manufacturing feasibility assessment

#### Fabrication feasibility

Print success was defined as complete implant fabrication without premature termination, severe warping (> 2 mm deviation from the build plate), or structural failure during support removal. The success rate was calculated as the ratio of usable implants to total printing attempts for each material. Active printing time was recorded from extrusion initiation to completion, excluding the 2.5-hour system preheating phase. Total workflow time was measured from CAD finalization to the sterilization-ready implant.

Post-processing characteristics were qualitatively evaluated during support removal, drilling operations, and surface finishing, with observations recorded regarding material behavior, tool interaction, and structural integrity. Dimensional stability was assessed using digital caliper measurements (± 0.01 mm precision) at designated reference points after 72 h of ambient storage.

Following post-processing, all implants were test-fitted to 3D-printed anatomical models (derived from the same CT dataset) by an experienced CMF surgeon (N.S.) to verify anatomical conformance, edge adaptation, and the absence of geometric interference. Fit quality was assessed qualitatively based on gap-free seating, contact continuity along the implant margins, and the absence of rocking or instability during placement.

#### Surface quality

Surface morphology was assessed using laboratory stereomicroscopy (Leica DFC 450 C, Wetzlar, Germany) at 31× magnification. Visual inspection examined layer adhesion quality, surface texture uniformity, and presence of defects (voids, delamination, or surface irregularities) across representative surface regions and cross-sections. Qualitative observations were documented with representative photomicrographs captured under standardized lighting conditions.

#### Dimensional accuracy

All successfully fabricated implants (*n* = 6 per design type) were digitized using high-resolution structured-light scanning with point accuracy of 4 μm (E4, 3Shape, Copenhagen, Denmark), both pre- and post-sterilization (Fig. 2I). Scan data were aligned to original CAD geometries using best-fit algorithms in mesh analysis software (Materialise 3-matic v.18, Materialise NV, Leuven, Belgium) (Fig. 2J).

Manufacturing accuracy was quantified as the root-mean-square (RMS) deviation and the median absolute deviation between the CAD reference and pre-sterilization scans. Critical feature dimensions (screw hole diameters, wall thickness, channel widths) were measured directly from scan data. For BCP-PEEK implants, porous surface regions generated during slicing were excluded from global deviation calculations as these features were not explicitly defined in the reference CAD geometry.

Dimensional stability following sterilization was assessed by comparing pre- and post-sterilization scan data using identical alignment protocols. Color-coded deviation maps were generated to visualize the spatial distribution of manufacturing errors and sterilization-induced changes (Fig. 2K).

## Results

### Fabrication feasibility

#### Print success and processing characteristics

**Fig. 4:**
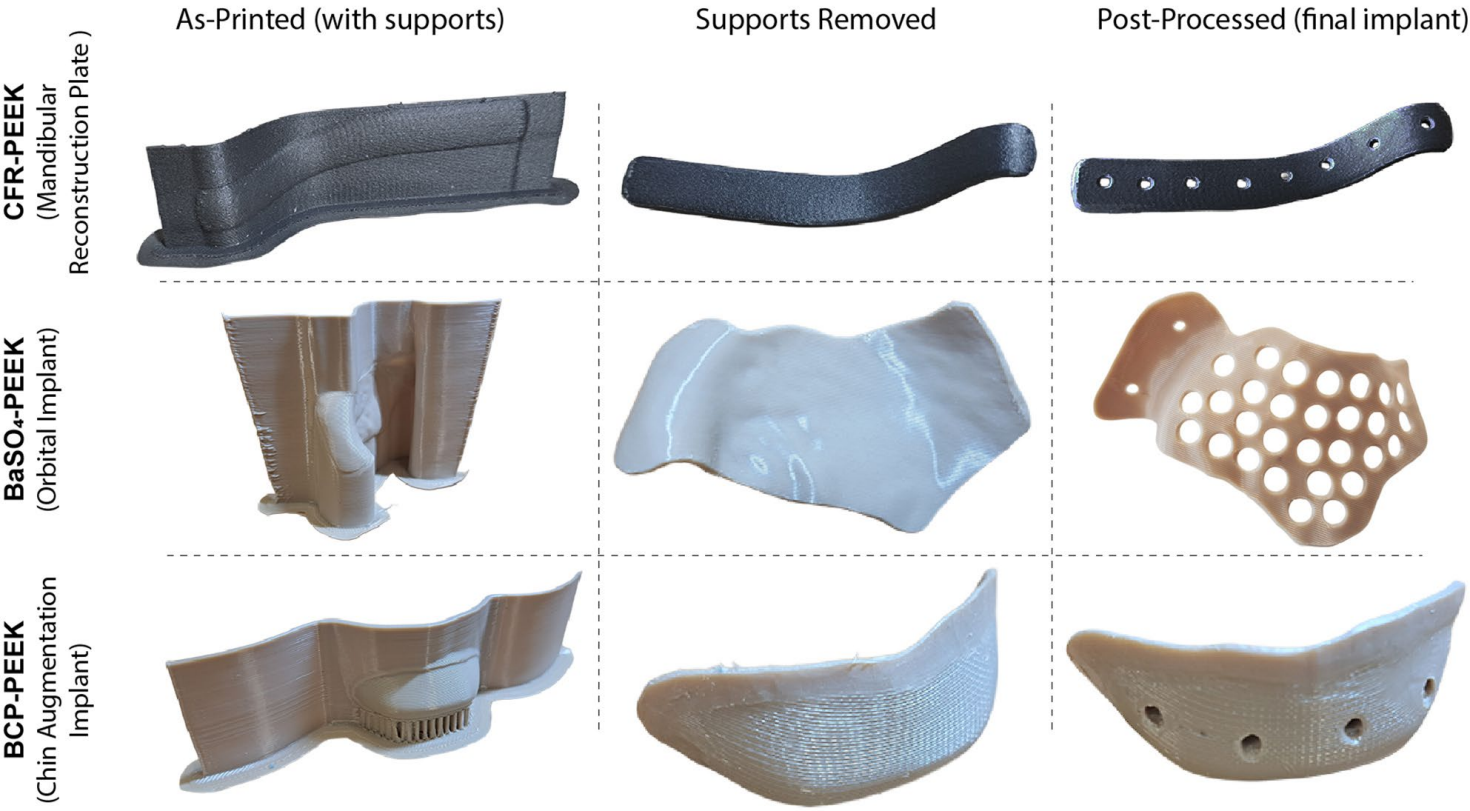
Manufacturing workflow progression for 3D-printed patient-specific implants fabricated from functionalized PEEK composites. Left to right: as-printed with supports, after support removal, and final post-processed state for CFR-PEEK mandibular reconstruction plate (top), BaSO₄-PEEK orbital implant (middle), and BCP-PEEK chin augmentation implant (bottom). The final column shows drilled fixation/drainage holes and finished surfaces

All three functionalized PEEK composites were successfully fabricated into patient-specific CMF implants (Fig. 4). Following parameter optimization, material-specific success rates were achieved: CFR-PEEK 100% (6/6 mandibular plates), BaSO₄-PEEK 100% (6/6 orbital implants), and BCP-PEEK 85.7% (6/7 chin implants, with one print excluded due to mid-print layer shift > 1.5 mm necessitating replacement).

CFR-PEEK exhibited increased filament brittleness during handling and elevated shear resistance during extrusion, though no nozzle clogging occurred when the material was dried correctly. The first-layer extrusion width was increased by 5% to compensate for initial underextrusion. BaSO₄-PEEK demonstrated processing behavior similar to that of unfilled, native PEEK, with predictable flow characteristics. BCP-PEEK maintained consistent quality across variable-density regions with no defects at solid-porous interfaces.

#### Production efficiency

Active printing time (excluding 2.5-hour preheating) averaged 30 min for mandibular plates, 41 min for orbital implants, and 58 min for chin implants. The complete workflow from CAD finalization to a sterilization-ready implant required 10–12 h, including post-processing. Material utilization efficiency (printed implant mass / total filament consumed) averaged 78–82% across all designs.

#### Post-processing characteristics

CFR-PEEK parts required controlled support removal due to increased rigidity, with standard carbide tools suitable, though they exhibited greater adhesion of molten material than unfilled PEEK. BaSO₄-PEEK exhibited excellent machinability, with easier support removal, though precise grinding control was required in thin-walled regions. BCP-PEEK demonstrated good machinability with slightly elevated surface hardness. No cracking or stress-induced failure occurred during finishing operations for any material.

Caliper measurements at defined reference points revealed < 0.1 mm dimensional variation after 72 h of ambient storage, indicating dimensional stability across all composites. All implants were confirmed for anatomical fit by clinical conformance prior to dimensional scanning.

### Surface quality

**Fig. 5:**
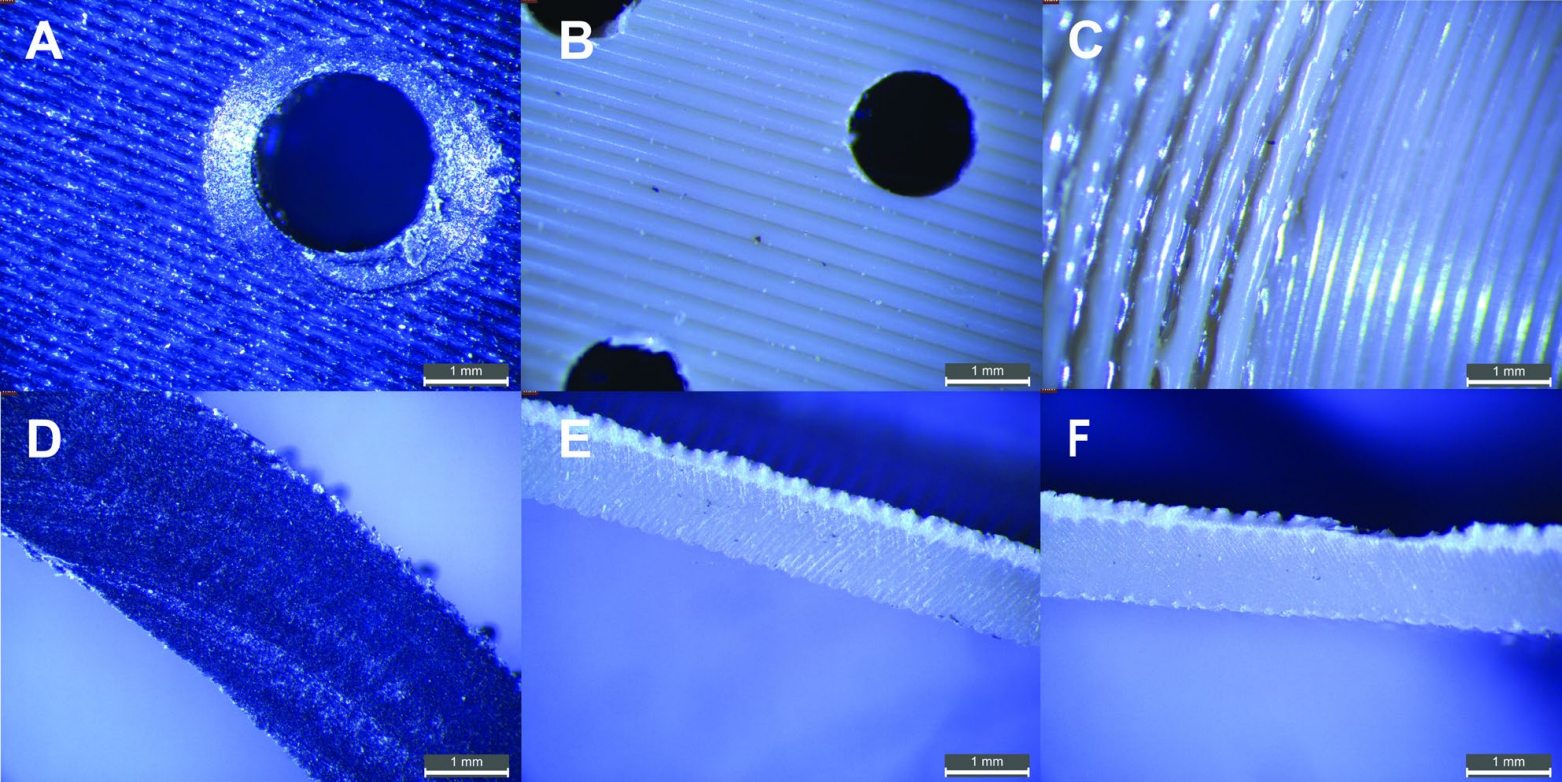
Microscopic characterization of implant surfaces and cross-sections. Representative stereomicroscope images showing (**A**) CFR-PEEK mandibular reconstruction plate surface, (**B**) BaSO₄-PEEK orbital implant surface, and (**C**) BCP-PEEK chin augmentation implant surface. Corresponding cross-sectional views for (**D**) CFR-PEEK, (**E**) BaSO₄-PEEK, and (**F**) BCP-PEEK implants. All images were acquired at 31× magnification with 1 mm scale bars

Surface quality assessment via stereomicroscopy revealed material-specific characteristics across all successfully fabricated implants. CFR-PEEK mandibular plates’ surface exhibited visible carbon fiber orientation at layer interfaces, resulting in a matte texture, particularly in shallow overhang regions. Interlayer adhesion was robust under manual stress testing. Stereomicroscopy revealed complete layer fusion with no observable voids or delamination in surface or cross-sectional examination (Fig. 5A and D). Enhanced structural rigidity from fiber reinforcement was evident during handling.

On the other hand, BaSO₄-PEEK orbital implant surfaces were smooth with consistent wall thickness and less pronounced, highly uniform layer lines (Fig. 5B). Support removal was more straightforward than CFR-PEEK, though precise grinding control was challenging due to the thin-walled geometry. No delamination, voids, or surface defects were observed in cross-sections (Fig. 5E). BCP-PEEK chin augmentation implants had surface characteristics resembling those of BaSO₄-PEEK, with a slightly textured finish at the density transition zones between the solid exterior and the porous bone-contacting surfaces (Fig. 5C). Porous regions maintained a designed texture while preserving interlayer adhesion. No evidence of ceramic particle settling or phase separation was observed. Cross-sections confirmed consistent layer bonding without delamination or cracking (Fig. 5F).

### Dimensional accuracy

#### Pre-sterilization manufacturing accuracy

**Fig. 6:**
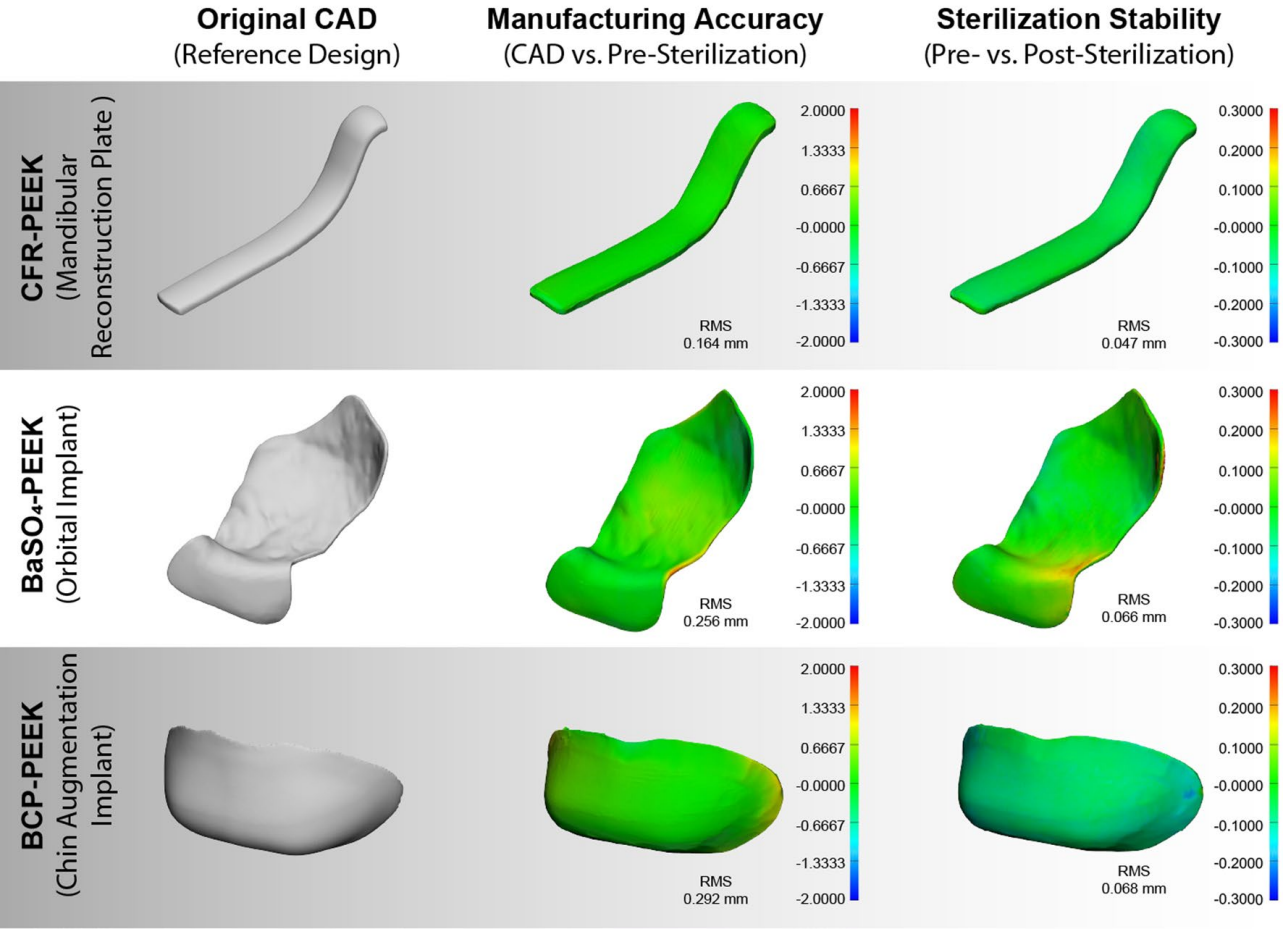
Dimensional accuracy evaluation. (Left) Original CAD reference designs. (Middle) Manufacturing accuracy: surface deviation maps comparing CAD to pre-sterilization scans (RMS deviations: CFR-PEEK mandibular reconstruction plate 0.16 mm, BaSO₄-PEEK orbital implant 0.26 mm, BCP-PEEK chin augmentation implant 0.29 mm). (Right) Sterilization stability: deviation maps comparing pre- and post-sterilization scans showing minimal dimensional changes (RMS 0.05–0.07 mm). Color scales represent deviation magnitude in millimeters

Dimensional comparison between fabricated implants (*n* = 6 per material) and CAD references revealed material-specific deviations (Fig. 6). CFR-PEEK mandibular plates demonstrated RMS deviation of 0.16 mm (median 0.02 mm). Despite the challenging double-curved spanning geometry (60–80 mm length), dimensional fidelity was maintained, with critical screw holes measuring 2.04 ± 0.03 mm, within the 2.00 mm design specification (+ 2% deviation), and within acceptable tolerances for bicortical fixation. The uniform 2.5 mm cross-section was preserved without warping across the entire span.

BaSO₄-PEEK orbital implants achieved an RMS deviation of 0.26 mm (median 0.01 mm). The thin-walled structure (0.80 mm nominal thickness) was reproduced with a measured thickness of 0.89 ± 0.02 mm (+ 11% deviation), indicating consistent slight overextrusion. Fine drainage channels maintained widths ≥ 1.5 mm. The complex three-dimensional orbital curvature was accurately captured despite the sub-millimeter wall thickness.

BCP-PEEK chin implants exhibited RMS deviation of 0.29 mm (median 0.05 mm) when excluding porous surface regions, which were procedurally generated during slicing rather than explicitly defined in the reference CAD geometry. The variable thickness gradient (7 mm central projection tapering to 0.6–0.8 mm peripheral edges) was successfully reproduced, with solid surface regions maintaining dimensional fidelity across spans up to 44 × 18 mm without warping or edge lifting.

Surgical test-fitting confirmed anatomical conformance for all implant types. All designs achieved gap-free seating with continuous edge-to-bone contact and no geometric interference, validating that the manufacturing accuracy was sufficient for clinical application despite geometry-specific dimensional variations.

#### Sterilization dimensional stability

Comparison of pre- and post-sterilization scans (*n* = 6 per material) revealed minimal dimensional changes following autoclave treatment. RMS deviations were 0.05 mm for CFR-PEEK, 0.07 mm for BaSO₄-PEEK, and 0.07 mm for BCP-PEEK (Fig. 6). These sterilization-induced changes were substantially minor than manufacturing deviations (0.16–0.29 mm RMS), representing < 30% of the initial manufacturing error for all composites. The negligible thermal effects confirm the dimensional stability of functionalized PEEK composites under standard steam sterilization protocols.

## Discussion

This proof-of-concept study demonstrates that three functionalized PEEK composites can be reliably processed into patient-specific CMF implants using high-temperature MEX 3D printing. The high fabrication success rates combined with clinically acceptable dimensional accuracy establish manufacturing feasibility as the technical foundation for subsequent validation phases [28–30].

Material-specific processing behaviors revealed critical practical considerations for clinical implementation. CFR-PEEK required substantially higher print speeds to minimize fiber-orientation artifacts while demanding careful handling due to its increased brittleness. The fiber texture at layer interfaces is both a material characteristic and a potential concern, as the literature documents the release of carbon fiber particles under mechanical stress and during surface-finishing operations. While short carbon fibers enhance mechanical properties, the risk of debris migration into surrounding tissues warrants a comprehensive biocompatibility assessment prior to clinical use, particularly with respect to inflammatory responses and long-term particle accumulation [31, 32]. Post-processing of CFR-PEEK required careful tool management to minimize fiber pullout during manual drilling of fixation holes, with tungsten carbide tooling required to handle the abrasive filler content.

BaSO₄-PEEK demonstrated the most predictable processing behavior with flow characteristics resembling those of unfilled PEEK despite 20% filler content, making it particularly suitable for delicate thin-walled geometries where process stability is critical. The chemically inert barium sulfate filler presents minimal biocompatibility concerns based on its established use in radiopaque medical devices, though its smooth surface finish, combined with its bioinert properties, may limit osseointegration potential without additional surface treatments [33, 34]. Post-processing proved straightforward, with manual drilling of drainage channels requiring careful control to prevent unintentional over-removal of material in thin-walled regions.

BCP-PEEK successfully processed variable-density architectures with seamless transitions between solid exterior surfaces and reduced-infill bone-contacting regions. The bioactive calcium phosphate phase aims to promote osseointegration through osteoconductivity [35], though the single-layer shift failure during extended print time indicates that process control for geometrically complex, time-intensive prints requires continued refinement. The ceramic filler introduced a slight increase in surface hardness during post-processing, though drilling the fixation hole remained feasible with standard carbide tooling.

The 10–12 h production timeline from design completion to a sterilization-ready implant represents a substantial acceleration compared to traditional external manufacturing workflows, which typically require weeks of vendor lead time [36]. This rapid turnaround may enable responsive surgical planning for trauma reconstruction, though the 2.5-hour system preheating requirement presents scheduling considerations for on-demand emergency manufacturing. The successful implementation of 3D-printed guides for the accurate positioning of manually drilled fixation and drainage holes demonstrates that workflow integration extends beyond the printing process itself.

The interpretation of dimensional accuracy reveals both achievements and opportunities for refinement. All materials maintained anatomical conformance validated by surgical test-fitting, confirming sufficient geometric fidelity for functional implants. The successful anatomical fit of the BaSO₄-PEEK orbital implant aligns with evidence demonstrating that patient-specific 3D models improve implant conformance compared to conventional free-hand shaping [37]. The systematic thickness deviation in orbital implants (+ 11% over design specification) represents controlled over-extrusion, suggesting that parameter adjustment through reduced nozzle flow rates could improve dimensional control while maintaining the excellent surface finish and structural integrity already achieved. It is important to distinguish between global RMS deviation and localized systematic deviations of this kind. While the observed over-extrusion did not impair anatomical fit during model-based test-fitting, sub-millimetre thickness variations in orbital implants warrant clinical attention, as they may influence implant seating on the bony ledge and soft tissue accommodation in anatomically constrained regions. Clinically meaningful tolerance thresholds for orbital implant accuracy have yet to be established in prospective studies that incorporate intraoperative fit assessment and postoperative volumetric outcomes.

Critical features created through post-processing, such as screw hole compatibility and drainage channel patency, depended on accurate printed geometry serving as substrate for manual finishing operations, confirming that as-printed dimensional fidelity translates to functional precision in final implants. The higher-dimensional variation in BCP-PEEK reflects challenges associated with variable thickness profiles and porous surface generation during slicing rather than explicit CAD definition, complicating direct geometric comparisons. The maintained anatomical fit despite these measurement artifacts supports their clinical applicability.

Minimal dimensional changes following steam sterilization confirm the thermal stability of all three composites under standard medical device sterilization protocols. This stability is vital for clinical workflow, as it enables implants to be manufactured, quality-controlled, sterilized, and stored without concerns for geometric deterioration [38, 39]. While comprehensive sterilization validation remains necessary for regulatory approval, these initial results indicate that functionalized PEEK composites retain sterilization compatibility with native PEEK.

The application-specific material selection strategy, matching mechanical reinforcement, radiographic visibility, and biological activity to anatomical requirements, enables optimized material-function alignment while maintaining patient-specific geometric customization. However, this proof-of-concept study has essential limitations that must be addressed before clinical translation. Quantitative functional validation remains a critical next step: three-point bending and compressive strength testing under simulated physiological loading are required for CFR-PEEK mandibular plates; quantitative CT and fluoroscopic contrast assessment across standard clinical imaging protocols is needed for BaSO₄-PEEK orbital implants; and in vitro osteoblast adhesion and proliferation assays are necessary for BCP-PEEK implants to confirm the expected bioactive response. Furthermore, the interaction between filler content, processing parameters, and printing-induced anisotropy may significantly influence mechanical and microstructural integrity, and warrants dedicated characterization in future work. Optical surface scanning confirmed external dimensional fidelity against the CAD reference; however, sub-surface porosity, incomplete interlayer fusion, delamination, and fibre distribution within the composite matrix remain uncharacterised. Micro-CT evaluation is therefore included as a priority objective in the planned follow-up characterization study, alongside mechanical testing and biological validation.

Future work should integrate these validation phases while exploring enhancements such as surface modifications to improve BaSO₄-PEEK osseointegration, coatings or sealants to mitigate CFR-PEEK particle release, and automated post-processing to reduce operator dependency. Longer-term opportunities include multi-material hybrid implants and integration with surgical navigation.

Economic considerations are an important practical factor for institutional adoption of POC PEEK composite manufacturing. The high-temperature MEX PEEK system used in this study represents a capital investment of approximately €215,000 (CHF200,000). Beyond equipment, significant one-time regulatory setup costs include biocompatibility testing, risk management documentation, clinical evaluation, and full production process validation prior to first clinical use. Ongoing operational costs encompass materials, consumables, maintenance contracts, quality system maintenance, and post-market surveillance. Staffing requirements, including a clinical lead responsible for surgical indication and design oversight, qualified clinical design engineers, AM technicians, and dedicated quality personnel, represent additional establishment and recurring costs. In practice, these roles may be fulfilled by a small team or, in some cases, by individuals combining clinical, engineering, and manufacturing competencies, a model that is particularly common in academic medical centers pioneering POC manufacturing. A formal cost-effectiveness analysis comparing POC composite PEEK production against externally manufactured alternatives remains an important objective for future work.

Beyond technical feasibility, sustainable POC manufacturing requires a compliance infrastructure that extends well beyond the implementation of a quality management system (QMS). In practice, this requires end-to-end workflow integration across imaging, segmentation, design, fabrication, post-processing, and documentation, with clearly defined responsibilities, digital traceability, and validated processes at each step. While an appropriate QMS aligned with ISO 13,485 provides the necessary structural framework under Article 5(5) of EU MDR 2017/745 [19], hospitals must additionally establish risk management per ISO 14,971, clinical evaluation including post-market surveillance, and biocompatibility testing per ISO 10,993 on samples processed through the complete production and sterilization pathway. Integrated functional systems for non-conformance investigation, corrective and preventive action, change control, and supplier qualification are further requirements that demand dedicated clinical, engineering, and quality expertise [18, 21]. Establishing and maintaining this infrastructure demands significant resources, specialized expertise, and dedicated personnel, underscoring the need to approach POC manufacturing as a long-term institutional commitment requiring governance structures, defined roles and sustainable funding.

The demonstrated manufacturing feasibility advances the vision of compliant POC innovation, where application-matched material properties can be tailored to specific clinical requirements while maintaining geometric precision demanded by individual patient anatomy.

## Conclusion

This proof-of-concept study demonstrates the technical feasibility of manufacturing application-specific PEEK composites for CMF reconstruction via high-temperature MEX 3D printing. CFR-PEEK, BaSO₄-PEEK, and BCP-PEEK were successfully printed into patient-specific mandibular, orbital, and chin implants with high production success and clinically acceptable dimensional accuracy. The systematic material-function matching, aligning mechanical reinforcement, radiographic visibility, and bioactivity to anatomical requirements, combined with rapid production capability, establishes fabrication feasibility for POC AM solutions. While comprehensive mechanical testing, radiographic validation, and biological assessment remain necessary for clinical translation, this work demonstrates that functionalized PEEK composites can be reliably produced into geometrically accurate, application-matched implants, advancing personalized reconstruction strategies in CMF surgery.

## Data Availability

No datasets were generated or analysed during the current study.

## Abbreviations

3D: Three-Dimensional
AM: Additive Manufacturing
BaSO_4_: Barium Sulfate
BCP: Biphasic Calcium Phosphate
CAD: Computer Aided Design
CFR: Carbon Fiber Reinforced
CMF: Craniomaxillofacial
CT: Computed Tomography
DICOM: Digital Imaging and Communications in Medicine
EU: European Union
GSPR: General Safety and Performance Requirements
ISO: International Organization for Standardization
LFD: Low Force Display
MDR: Medical Device Regulation
MEX: Material Extrusion
PEEK: Polyetheretherketone
PLA: Polylactic Acid
POC: Point of Care
PSI: Patient-Specific Implant
RMS: Root Mean Square
SLA: Stereolithography
STL: Standard Tessellation Language
